# Sensor‐mediated granular sludge reactor for nitrogen removal and reduced aeration demand using a dilute wastewater

**DOI:** 10.1002/wer.1296

**Published:** 2020-02-05

**Authors:** Zerihun A. Bekele, Jeseth Delgado Vela, Charles B. Bott, Nancy G. Love

**Affiliations:** ^1^ Department of Civil and Environmental Engineering University of Michigan Ann Arbor Michigan; ^2^ Department of Civil and Environmental Engineering Howard University Washington District of Columbia; ^3^ Hampton Roads Sanitation District Virginia Beach Virginia

**Keywords:** aeration control, mainstream N removal, NOB suppression, partial nitritation/anammox

## Abstract

A sensor‐mediated strategy was applied to a laboratory‐scale granular sludge reactor (GSR) to demonstrate that energy‐efficient inorganic nitrogen removal is possible with a dilute mainstream wastewater. The GSR was fed a dilute wastewater designed to simulate an A‐stage mainstream anaerobic treatment process. DO, pH, and ammonia/nitrate sensors measured water quality as part of a real‐time control strategy that resulted in low‐energy nitrogen removal. At a low COD (0.2 kg m^−3^ day^−1^) and ammonia (0.1 kg‐N m^−3^ day^−1^) load, the average degree of ammonia oxidation was 86.2 ± 3.2% and total inorganic nitrogen removal was 56.7 ± 2.9% over the entire reactor operation. Aeration was controlled using a DO setpoint, with and without residual ammonia control. Under both strategies, maintaining a low bulk oxygen level (0.5 mg/L) and alternating aerobic/anoxic cycles resulted in a higher level of nitrite accumulation and supported shortcut inorganic nitrogen removal by suppressing nitrite oxidizing bacteria. Furthermore, coupling a DO setpoint aeration strategy with residual ammonia control resulted in more stable nitritation and improved aeration efficiency. The results show that sensor‐mediated controls, especially coupled with a DO setpoint and residual ammonia controls, are beneficial for maintaining stable aerobic granular sludge.

**Practitioner points:**

- Tight sensor‐mediated aeration control is need for better PN/A.
- Low DO intermittent aeration with minimum ammonium residual results in a stable N removal.
- Low DO aeration results in a stable NOB suppression.
- Using sensor‐mediated aeration control in a granular sludge reactor reduces aeration cost.

## Graphical abstract

Multiple metabolic pathways and competition for nitrite exist in the treatment of anaerobically pretreated mainstream wastewater using a granular sludge reactor.

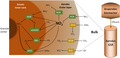

## Introduction

there is great interest in wastewater systems that are energy neutral or positive to also achieve resource recovery using means that meet stringent effluent standards. The A‐B (adsorption–biooxidation) process targets this end point. The A‐stage is dedicated to maximizing carbon capture for later energy production and is commonly deployed using high‐rate activated sludge (HRAS) (Jimenez et al., 2015) or chemically enhanced primary treatment (CEPT) (Diamantis et al., 2013). Recently, the anaerobic membrane biofilm reactor (AnMBR) has been proposed as a viable A‐stage technology that could become an energy‐efficient option (Smith et al., 2014). The B‐stage is focused on energy‐efficient nutrient (commonly nitrogen) management (Jetten, Horn, & van Loosdrecht, 1997; Wan, Gu, Zhao, & Liu, 2016). Most of the energy expense in an A‐B process occurs due to aeration in the B‐stage, where the remaining carbon and nitrogen (N) are removed. In some cases, it is possible for the energy expense in the B‐stage to negate the energy gained in the A‐stage, making A‐B process inefficient for energy recovery (Zhou et al., 2013).

Processes that require less oxygen and do not require external substrate are desirable to minimize aeration demand in the B‐stage. Traditionally, N removal is done via complete nitrification (first by ammonium‐oxidizing bacteria [AOB] and then by nitrite‐oxidizing bacteria [NOB]) followed by heterotrophic denitrification via ordinary heterotrophic organisms (OHOs). This process has a high aeration demand and requires a higher theoretical oxygen demand (ThOD)‐to‐nitrogen (ThOD/N) ratio (>5) compared to other novel processes (Daigger, 2014). In particular, A‐stage processes such as HRAS and CEPT are less efficient at removing nitrogen (de Graaff et al., 2016; Miller et al., 2016), resulting in an effluent ThOD/N ratio <5. This ratio is insufficient for N removal, because additional carbon will be lost during aeration for nitrification. In this case, exogenous electron donor may be needed to achieve high levels of N removal.

Compared to HRAS and CEPT, the AnMBR A‐stage process may be better suited to achieve an overall A‐B process energy efficiency. The effluent from mainstream anaerobic treatment typically contains organic carbon (45–145 mg COD/L), ammonium (19–53 mg N/L), dissolved methane (40–140 mg ThOD/L), and sulfide (0–145 mg ThOD/L) (Delgado Vela et al., 2015). Hence, if we consider dissolved methane, sulfide, and ammonium as potential electron donors in addition to organic carbon for N removal in a downstream B‐stage system, it will be enough for complete N removal. Methane and sulfide in the AnMBR effluent are possible electron donors for N removal with nitrite/nitrate as electron acceptors via denitrifying anaerobic methane oxidation (DAMO) (Raghoebarsing et al., 2006) and sulfide oxidation (Souza & Foresti, 2013). Among these options, pursuing N removal via nitrite is preferred as it uses less electron donor and less aeration for nitritation. Ammonia can be used as electron donor for anaerobic ammonia oxidation (anammox), which requires nitrite as an electron acceptor (Strous, Jetten, Heijnen, & Kuenen, 1998). However, any of these approaches require an operational strategy that reliably allows nitritation and minimizes loss of electron donors. Assuming this can be done, then partial nitritation and anammox (PN/A) become the most attractive B‐stage N removal option, as its low aeration demand and no organic carbon demand (Winkler, Kleerebezem, & Loosdrecht, 2012).

**Figure 1:**
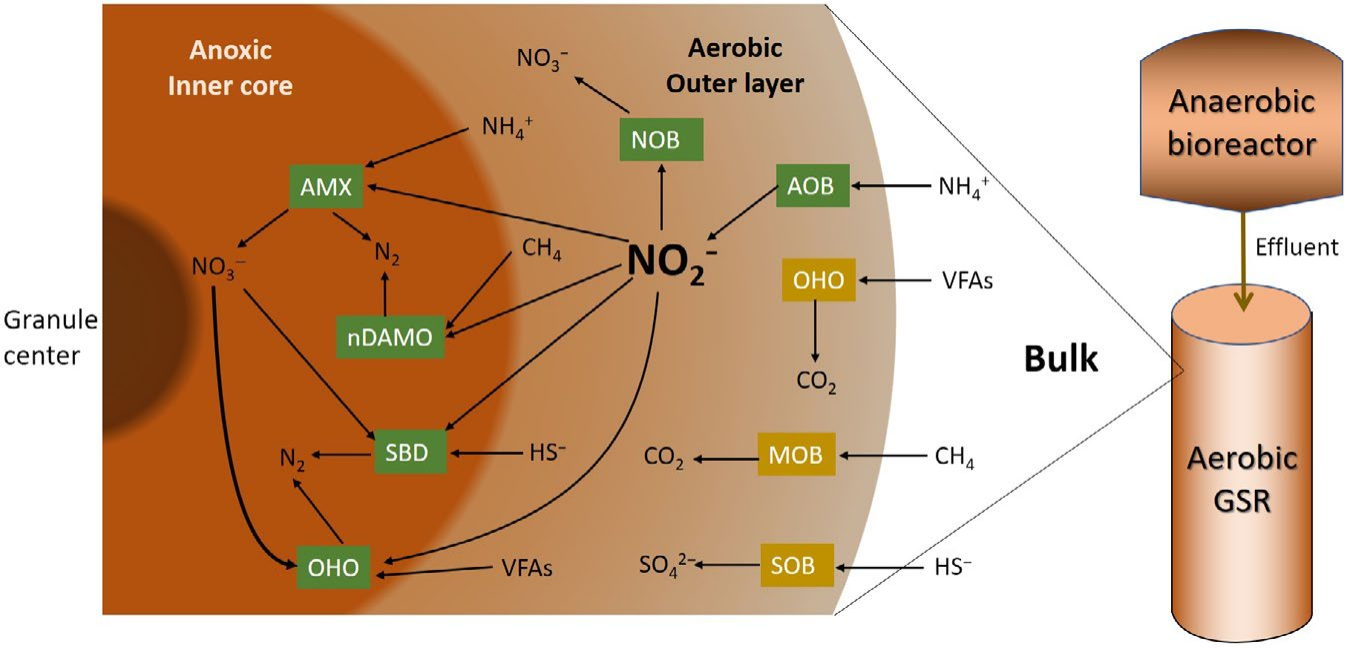
Potential metabolic pathways in a B‐stage GSR for removal of ammonia, VFA, methane, and sulfide present in an anaerobically treated A‐stage. MOB, methane‐oxidizing bacteria; SBD, sulfur‐based denitrification; SOB, sulfur‐oxidizing bacteria.

Besides targeting efficient N removal processes, the use of advanced biofilm systems such as aerobic granular sludge reactor (GSR) as a prospective B‐stage technology has significant advantages over conventional activated sludge systems (Sarma, Tay, & Chu, 2017). Noted advantages of aerobic GSR include the presence of different redox zones within the granules that support a diverse microbial ecology; high rates of settleability; and a high biomass retention which is ideal for slow‐growing N‐removing bacteria (de Kreuk, Heijnen, & van Loosdrecht, 2005; Liu, Yang, & Tay, 2003; Szabó et al., 2017). Figure 1 shows the potential key microbial groups and their interactions during complete nitrogen removal via nitrite in a single stage GSR downstream of an AnMBR A‐stage. The schematic emphasizes that competition for nitrite will occur among different anaerobic organisms, and suggests that successful N removal requires sustained partial nitritation while suppressing nitrite oxidation by NOB. Therefore, the success of an energy‐efficient aerobic GSR as a B‐stage N removal system requires a robust operating strategy that favors simultaneous partial nitritation and denitritation by suppressing NOB.

One of the main challenges for N removal in mainstream wastewater via nitrite is suppression of NOB. In particular, the suppression of NOB in PN/A process has been demonstrated at full‐scale for concentrated sidestream applications, which have favorable conditions such as low C/N ratio (<2 g COD/g N); high temperature (20–30°C); high free ammonia concentrations (>0.1 mg N/L) (Philips, Laanbroek, & Verstraete, 2002); and high (>0.2 mg N/L) free nitrous acid (Kornaros, Dokianakis, & Lyberatos, 2010). Unfortunately, most of these conditions are atypical for dilute mainstream systems (Cao, van Loosdrecht, & Daigger, 2017). However, if a sequencing batch reactor (SBR) is used and a minimum residual ammonium concentration (RAC) is maintained throughout the reaction time, NOB suppression can still be achieved via free ammonia that is present in sufficient concentration during most of the reaction cycle. Nonetheless, since this alone may not be enough to effectively suppress NOB, new strategies are needed for NOB suppression. For this reason, we propose the use of real‐time sensor‐mediated control (SMC) for robust aeration control to suppress NOB. Online sensors have been used to suppress NOB in sidestream applications by manipulating the DO setpoint or by using intermittent aeration and ammonium‐based aeration control (ABAC). For example, Regmi et al. (2014) used real‐time ABAC with intermittent aeration to suppress NOB in a suspended culture for mainstream nitritation. Lemaire, Marcelino, and Yuan (2008) used DO and pH sensors to control aerobic duration for shortcut N removal by suppressing NOB. Both studies demonstrate that SMC for mainstream NOB suppression is a viable option.

A‐stage effluent has a low organic load in concert with a much lower N load than sidestream granular systems, which can tolerate low organic loads given the high N loading (Wett et al., 2015). The combination of low organic and N loading with a low C/N ratio in dilute mainstream A‐B applications makes it challenging to develop and sustain granules. Tay, Pan, He, and Tay (2004) reported that they were not able to produce granules when the organic loading was below 2 kg COD m^−3^ day^−1^. The lowest organic loading rate that we found reported to date for successful granule formation came from Ni et al. (2009) and Zhang, Zhang, and Yang (2015) who used 0.6–1 and 0.37–0.56 kg COD m^−3^ day^−1^, respectively. Hence, developing stable granules in low‐loaded circumstances is another challenge that must be addressed to advance the GSR technology.

In this study, we focused on developing and operating an aerobic GSR as an exemplary B‐stage N removal system for an A‐stage AnMBR effluent. We use this reactor configuration to develop and demonstrate an SMC strategy that supports NOB suppression and reduces aeration energy. We evaluated the degree to which the strategy supports PN/A for N removal, and highlight the conditions needed to support stable granule formation.

## Methods

### Reactor setup

A glass bubble column reactor with 76.2 mm (3 inches) diameter and 711.2 mm (28 inches) height with a working volume of 4.5 L was operated for 474 days. The reactor was initially inoculated by mixing a nitrifying activated sludge from the Ann Arbor Wastewater Treatment Plant (Michigan, USA) and biomass from a full‐scale deammonification (DEMON) unit (Hampton Roads Sanitation District, Virginia, USA). The reactor was fed with a simulated mainstream anaerobic digester effluent containing ammonium (48 ± 6 mg/L‐N), VFAs (acetate and propionate, 100 mg/L‐COD), dissolved methane at saturation (~22 mg/L‐CH_4_), and other trace elements. Details of the media preparation procedure can be found in the Supporting Information Appendix S1: Section 12. The C/N ratio, considering VFA and ammonium, was from 1.85 to 2.5. The reactor was monitored and controlled using online optical DO (WTW; FDO 925; Xylem Inc.), pH (accumet® Electrode; Fisher Scientific), and ${\text{NH}}_{4}^{+}/{\text{NO}}_{3}^{-}$ (IQ SensorNet VARiON® Plus Sensors; Xylem Inc.) probes to suppress NOB and favor the growth of AOB and anammox species. A bulk DO concentration was held at specific DO levels between 0.2 and 1.5 (±0.1 mg/L O_2_). The pH was monitored using an online probe and was maintained between 7.3 ± 0.2 and 8.0 ± 0.2 by dosing NaHCO_3_. The entire experiment was conducted at an ambient temperature between 20 and 23°C.

### Cycle operation

The reactor was operated in a sequencing batch mode with a 40‐min anoxic slow feed from the bottom of the reactor, followed by intermittent aeration with anoxic and aerobic cycles for 220–300 min, a settling time of 4–10 min, and 4 min of decanting with a volumetric exchange ratio of 50%. Air was supplied using a glass diffuser with a superficial upflow velocity of 1.6 cm/s. During the anoxic phase, gas from the head space of the reactor was pressurized by diaphragm pump, blended with dinitrogen gas and recirculated through the reactor. This supported the development of anoxic conditions and resupplied stripped methane gas to enhance the chance for its dissolution and metabolism. During the aerobic phase, a mixture of dry air, dinitrogen gas, and head space gas were pumped into the reactor. The dry air flow rate was controlled with a mass flow controller (MFC) device to maintain the desired setpoint using in‐house developed partial differential and integral (PID) controller (developed in LabVIEW® software).

### Sensor‐mediated control development

Across the aerobic phases of a single SBR cycle, DO was controlled in the first two phases based on a specific setpoint, and ammonia‐based aeration control was used for the last phase of operation. Both aeration schemes were implemented by developing a PID controller in LabVIEW (Supporting Information Appendix S1: Figure S5). The developed LabVIEW SMC program was designed to allow time‐based aeration for DO setpoint only and ABAC with a DO setpoint. The program also controls all the pumps and sensor‐based devices, thus automating the entire operation. Information reported as sensor‐derived concentration was adjusted based on correction factors determined by using simple linear regression between sensor output and analytically determined concentrations (i.e., for ammonium and nitrate). Effluent nitrite corrections reported in Figure 5 only were derived from its correlations with effluent ammonium and nitrate.

### Long‐term reactor operation

The reactor was operated for 474 days, which can be broken down into four phases (Supporting Information Appendix S1: Table S2). During the first phase (days 1–60), granule development occurred and the reactor was operated at a DO setpoint of 1.5 mg/L. A lower DO setpoint (0.5 mg/L) without ABAC was used during the second phase (days 61–200). The third phase (days 201–410) was operated at a DO setpoint of 0.75 mg/L without ABAC. The final and fourth phase (days 411–474) was operated with low DO setpoint (0.5 mg/L) and ABAC. Ammonium‐based aeration was implemented to maintain residual ammonium at or above 5 mg/L as N, both to promote anammox activity and to suppress NOB as indicated by Cao et al. (2017). During all phases, samples were usually collected three times a week from the reactor after settling using a sampling port and analyzed for ammonium (Standard Method [SM] Sec. 4500‐NH_3_ F), nitrite (SM Sec. 4500‐NO_2_^−^ B), nitrate (SM Sec. 4110 B), and methane (SM 2720, 6211, and 6010). Biweekly cross‐cycle sampling was done over a single operating cycle to obtain profiles of soluble N species, also volatile fatty acids (VFAs) using ion chromatography as described in Smith, Skerlos, and Raskin (2013). Additionally, in situ batch activity tests were conducted for phases 2, 3, and 4 to determine nitrification and anammox activities. The detailed procedure for this can be found in Supporting Information Appendix S1: Section 13. Physical characteristics of granules, such as size, sludge volume index (SVI), and solid retention time (SRT), were also monitored as described in the Supporting Information Appendix S1. All sensor data (i.e., ${\text{NH}}_{4}^{+}$, ${\text{NO}}_{3}^{-}$, DO, and pH) and other operation information were logged every minute. All values errors are given at a 95% confidence interval from a *t* test distribution.

### Microbial community analysis

Biomass samples were taken during the first two months of the reactor granulation phase and also at later stages of operation to monitor the microbial composition using 16S rRNA gene amplicon sequencing. The inoculum used to obtain an anammox organism was from a full‐scale deammonification unit at Hampton Roads Sanitation District (HRSD), Virginia. DNA was extracted using three bead‐beading steps followed by extraction with a Maxwell 16 LEV automated nucleic acid extractor (Promega, Madison, WI) using DNA blood kits. Amplicon sequencing of the V4 region of the 16S rRNA gene was performed on the Illumina MiSeq (MiSeq Reagent Kit V2 500 cycles; Illumina Inc., San Diego, CA) platform using the previously developed dual‐indexing sequencing strategy (Kozich, Westcott, Baxter, Highlander, & Schloss, 2013). Additional details of the procedure can be found in Delgado Vela, Dick, and Love (2018). Post‐processing of the Illumina MiSeq data was done using the Mothur MiSeq SOP (Schloss et al., 2009) without rarefication and including archaea in the analysis. Data from the MiSeq analysis have been uploaded to NCBI and are openly available under accession number PRJNA549919 (Bekele, 2019).

## Results

### Phase 1: Granule formation

**Figure 2:**
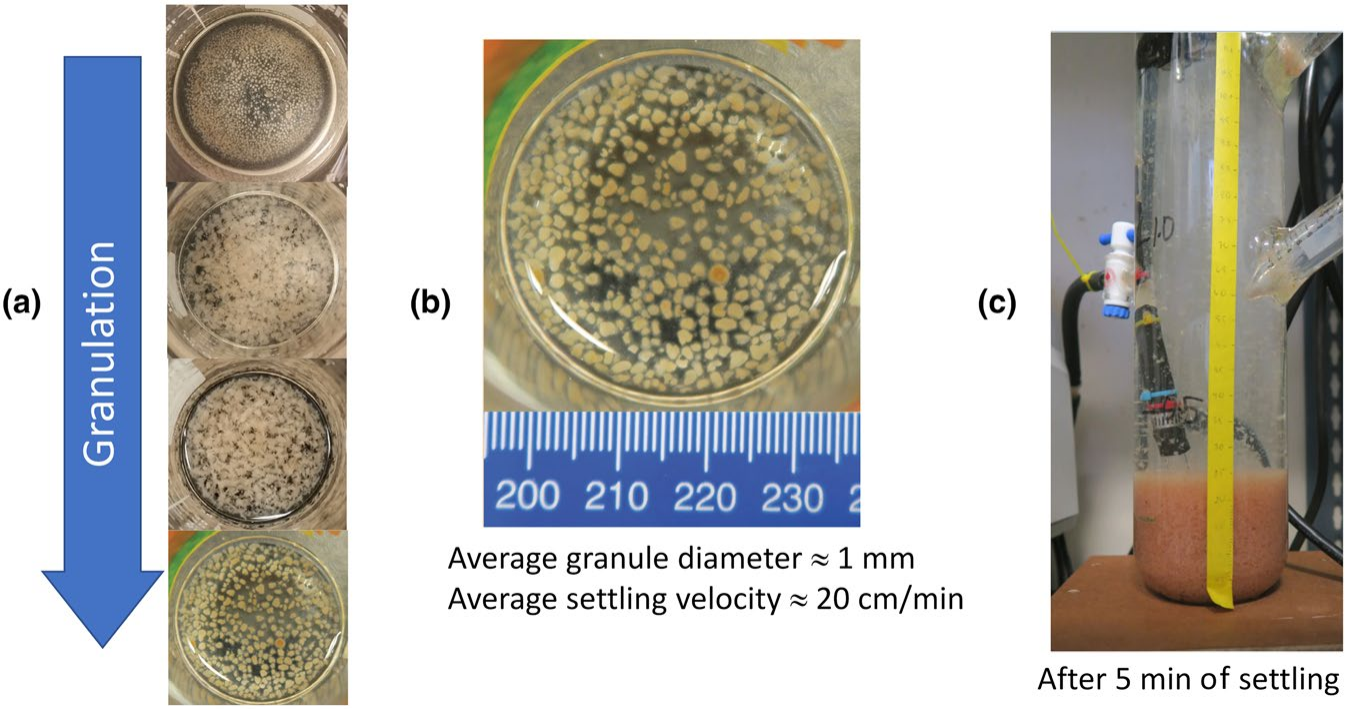
(a) Granules change over time during start‐up Phase 1. They began as micro‐granules, then became large and fluffy, and finally developed into mature granules. (b) Mature granules with an average size of 1 mm. (c) Granules in the reactor after 5 min of settling.

The initial phase of granule development took about 60 days (Figure 2), which is relatively rapid compared to other granular systems (Ni et al., 2009). For granule development and selection, the reactor was operated in SBR mode with a short (5 min) settling time and a superficial upflow velocity of 1.6 cm/s. The granules went through different morphological transformations as they were formed (Figure 2). First, micro‐granules began to form after 2 weeks of operation. Then, large and fluffy granules formed after a month. Finally, the granules developed into mature granules after two months of operation. The mature granules had an average diameter of 0.97 ± 0.06 mm (Supporting Information Appendix S1: Figure S2) and an average settling velocity of 12 m/hr. The steady‐state mean VSS concentration was 1,520 ± 176 mg/L with a mean 5 min SVI of 70 mg/L. We calculated an average SRT of 4.8 ± 0.6 days, 12.2 ± 2.9 days, 11.9 ± 2.1 days, and 17.5 ± 3.5 days for Phase 1, Phase 2, Phase 3, and Phase 4, respectively (Supporting Information Appendix S1: Figure S1).

**Figure 3:**
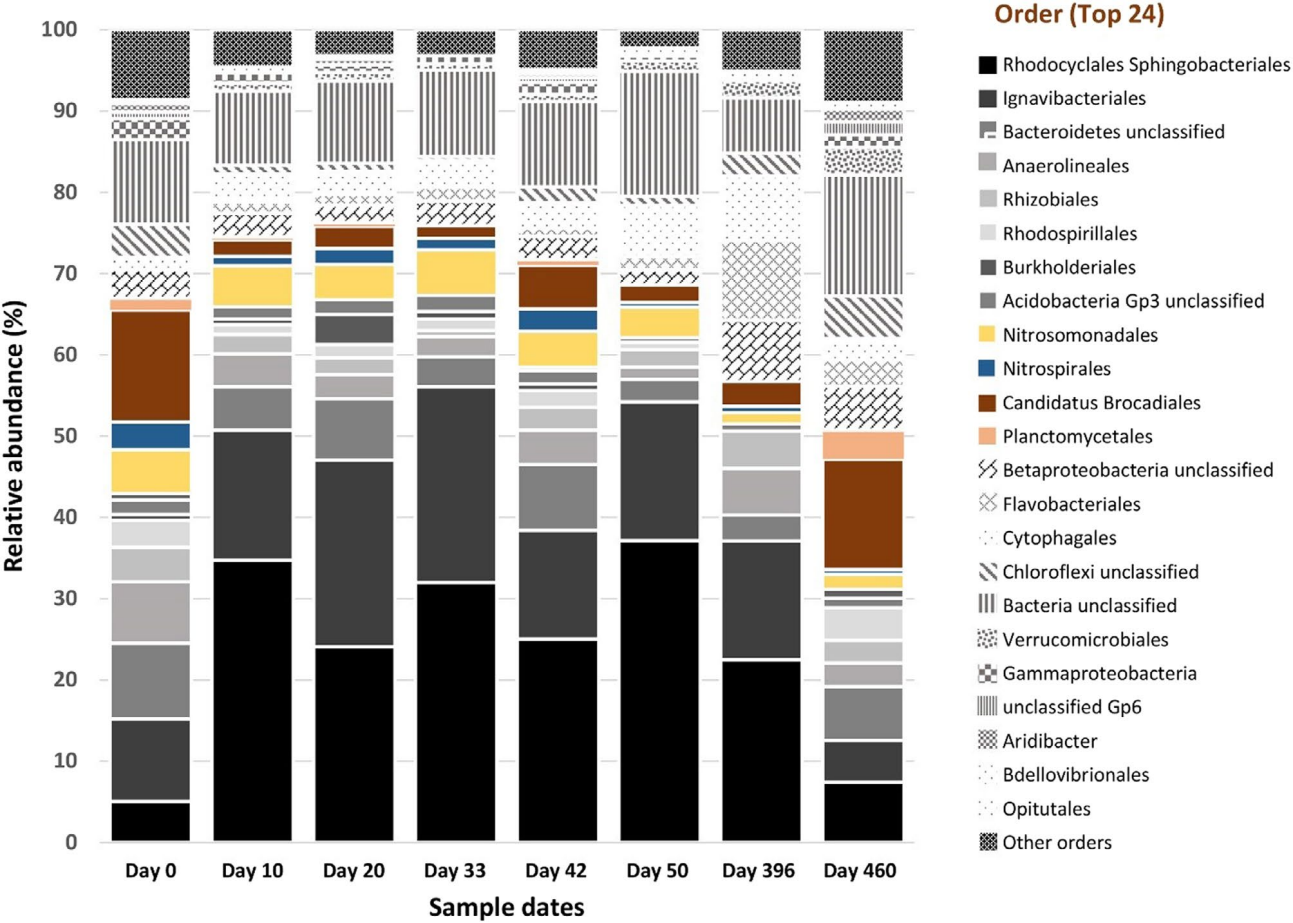
Microbial composition dynamics at order‐level OTUs for the period of granule development (through day 50), day 396 which is in Phase 3, and day 460 which is in Phase 4. Solid graytone colors are OHOs, hatched graytones are other bacteria with either known or unknown functions such as EPS production, hydrolysis, and filament formation, and solid nongraytone colors are AOB, NOB, anammox, and the order planctomycetales.

**Figure 4:**
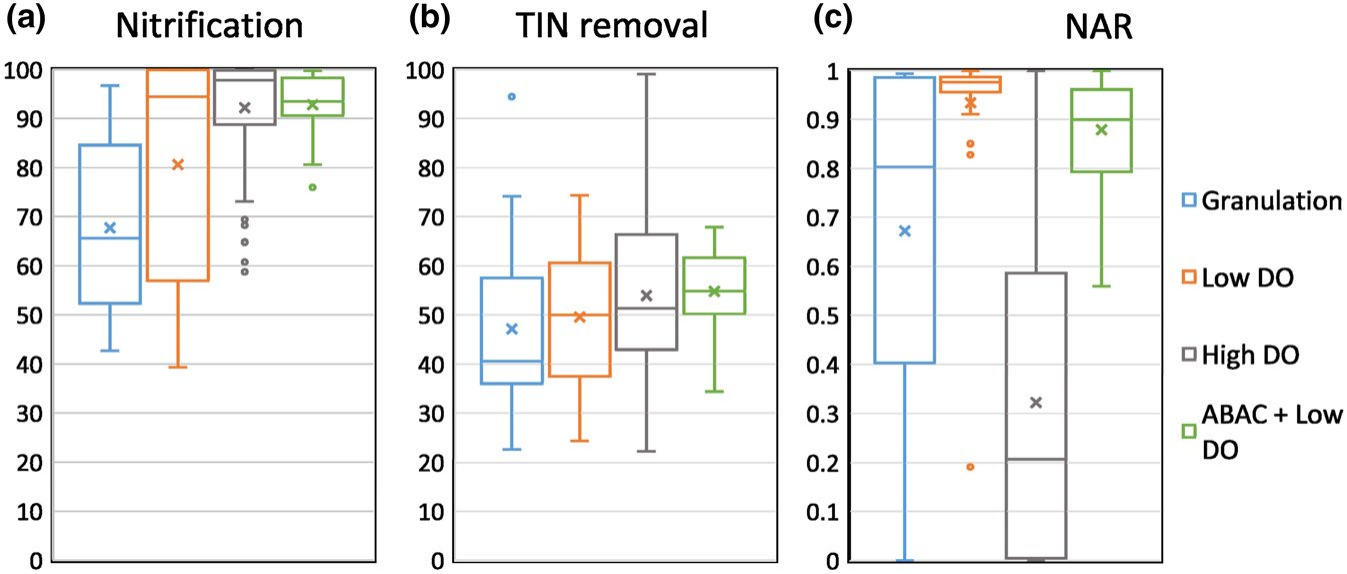
Boxplots showing comparisons across the four operation stages for (a) percent nitrification, (b) percent total inorganic nitrogen removal, and (c) nitrite accumulation ratio (NAR = effluent nitrite‐N:effluent [nitrite‐N + nitrate‐N]). Note: “*x*” indicates the mean, and the inside horizontal line indicates the median.

During granulation, the biomass color changed from dark red (inoculum, not shown) to pale yellow (Figure 2), indicating a shift in microbial composition. Whole community analysis based on the 16S rRNA gene was used to characterize how the microbial community shifted over the course of reactor operation, including during the granulation period (Figure 3). Community analysis showed that the only anammox (AMX) taxa detected in the inoculum were of the order “*Candidatus Brocadiales*,” and comprised approximately 13% of the community. Subsequently, the relative abundance of AMX decreased to 2.2% by the end of granulation. During this same period, nitrite and ammonium accumulated while nitrate was mostly absent. Although substrates required for anammox metabolism were present, the loss of AMX suggests that this metabolism was not occurring to a significant degree. Concurrently, the inoculum contained relatively equal fractions of AOB (genus *Nitrosomonas*, 3.7%) and NOB (genus *Nitrospira*, 3.4%); however, by the end of the granulation period AOB had a higher relative abundance (5.4%) than NOB (0.6%), consistent with NOB out‐selection. The nitrite accumulation rate (NAR = 0.67 ± 0.24) was consistent with this result (see Figure 4). Furthermore, OHOs increased from 20% to 50% over this same period; however, we presume that insufficient organic carbon was available as an electron donor to fully consume the accumulated nitrite as it was observed in Phase 2 and Phase 3 (Supporting Information Appendix S1: Figure S6). These results suggest that a rapid, or aggressive, granulation period makes it difficult to establish stable redox niches that are needed to support slow‐growing microorganisms, such as AMX and NOB. Furthermore, the high DO of 1.5 mg/L used during the aerobic period might have reduced the size of the anoxic zone in the granules, which may have limited AMX activity. Collectively, these factors all likely contributed to the reduction in N removal during the granulation phase.

### Phase 2: Low (0.5 mg/L) dissolved oxygen operational phase

In Phase 2 operation when the bulk DO setpoint was 0.5 mg‐O_2_/L, nitrite was routinely present in the effluent at an average concentration of 13.9 ± 3.4 mg N/L while nitrate averaged 0.4 ± 0.2 mg N/L with high NAR of 0.93 ± 0.06 (Figure 4). The effluent ammonium concentration was 8.4 ± 3.5 mg N/L and quite variable (Supporting Information Appendix S1: Figure S4), ranging between 25 and below detection (seven of 25 measurements during this phase were at or below 0.1 mg/L as N). The overall TIN removal during this phase was 49.6 ± 5.6%, and the overall ammonia conversion was 80.7 ± 8.1% (Figure 4). This broad range of performance during Phase 2 may have made it difficult to sustain significant anammox growth in the system.

To estimate of the relative contributions of AMX and OHOs to TIN removal, we used cross‐cycle data (i.e., one batch cycle N and VFA profile data) with theoretical stoichiometric equation (see Supporting Information Appendix S1: Section 10, from Tables S9–S16). The VFA data show that at least one‐third was utilized by the end of the first anoxic period and was consumed at the same time as residual nitrite held over from the prior cycle was consumed, implying that nitrite served as the electron acceptor during that period. The remaining VFA was rapidly oxidized during the first aerobic period. After accounting for nitrogen consumption for cell growth, our calculations suggest that TIN removal via OHOs occurred up to the end of the first anoxic period, and for the rest of the cycle via anammox. Our stoichiometric predictive analysis shows that VFA removed up to 72% of the oxidized inorganic nitrogen on average during Phase 2. We did not detect residual methane at the end of the anoxic feed period (Supporting Information Appendix S1: Figure S7) nor did we detect DAMO through our microbial community analysis; therefore, it is reasonable to assume that methane was stripped out as soon as mixing started in the first anoxic zone. Considering all these factors leaves an unaccounted source of TIN removal of at least 28%, which we assume is attributed to anammox during this phase. Consequently, despite its low relative abundance, AMX may have contributed to TIN removal.

In situ nitrification and anammox activity results from tests conducted during Phase 2 suggest that NOB were suppressed and AMX was active, corroborating the stoichiometric predictive analysis. AOB (0.32 g N‐NO_2_^−^ formed per g VSS per day) were 4 times more active than NOB (0.08 g N‐NO_3_^−^ formed per g VSS per day), even though DO exceeded 1.0 mg/L (Supporting Information Appendix S1: Table S6). The Phase 2 in situ anammox activity test yielded a specific total inorganic nitrogen (ammonium + nitrite) utilization rate of 0.104 mg‐N per mg‐VSS per day, supporting our prior conclusion that anammox was likely actively involved in TIN removal (see Supporting Information Appendix S1: Table S3).

### Phase 3: high (0.75 mg/L) dissolved oxygen operational phase

Phase 3 was operated at a higher bulk DO setpoint of 0.75–1 mg‐O_2_/L and resulted in both a sustained loss of nitrite and increase in nitrate. During this phase, effluent nitrite was present at an average concentration of 4.4 ± 1.1 mg N/L, while the effluent nitrate increased to an average concentration of 14.7 ± 2.6 mg N/L, which resulted in low NAR of 0.33 ± 0.08 (Figure 4). The effluent ammonium concentration was 4.0 ± 1.5 mg N/L with an overall conversion rate of 92 ± 3%, which is higher than in Phase 2 (*p* < 0.001). The overall TIN removal for this phase was 53.2 ± 4.1%, which is similar to the performance of Phase 2 (*p* > 0.05).

Cross‐cycle analysis showed that AMX and OHO both continued to contribute to TIN removal. VFA was predominantly consumed during the anoxic feed period via heterotrophic denitrification that used the residual nitrate from the prior cycle (see Supporting Information Appendix S1: Figure S6). The remaining VFA was oxidized rapidly during the first aerobic period. Consequently, any TIN removal observed after the first aerobic period was assumed to be due to AMX. Based on cross‐cycle data (Supporting Information Appendix S1: Tables S17 through 24) and after accounting for N loss for growth, we estimate that VFA could, at best, remove 66% of the oxidized inorganic nitrogen present in the influent. Since AMX is present in the system and exposed to intermittent anaerobic periods, and a persistent anaerobic inner core exists in the granules despite a measurable bulk DO, we conclude that AMX removed up to 34% of TIN.

In situ nitrification and anammox activity tests indicated that NOB were more active than AOB, and anammox activity was detected but lower than what was measured during Phase 2 (see Supporting Information Appendix S1: Tables S3 and S6). The in situ nitrification test conducted on day 376 showed that the NOB activity rate (0.32 g N‐NO_3_^−^ formed per g VSS per day) was at least 3 times more active than AOB (0.099 g N‐NO_2_^−^ formed per g VSS per day), indicating that the NOB suppression observed during Phase 2 was reversed. In addition, the in situ anammox activity test conducted on day 362 showed that AMX had a specific total inorganic nitrogen utilization rate of 0.08 mg‐N per mg‐VSS per day, indicating that AMX was active but at a lower rate than what was measured during Phase 2. As a confirmation, we estimated the average rate of the net TIN oxidized by AMX only during Phase 3 to be 0.06 mg‐N per mg‐VSS per day from the cross‐cycle data. Since the in situ activity tests show the highest rate achievable under ideal conditions, this comparison shows that the anammox activity measured can explain the loss of oxidized TIN residual during a single cycle.

Microbial community analysis produced results consistent with the in situ activity experiments. Illumina MiSeq results from samples collected on day 396 (toward the end of Phase 3) indicate that the granules continued to contain AMX, AOB, and NOB but with a lower relative abundance compared to the granulation phase. In addition, granule size did not significantly change during Phase 2 (0.97 ± 0.05 mm) and Phase 3 (0.92 ± 0.02 mm) (two‐tail *t* test *p* = 0.1). This suggests that the shift in performance and establishment of nitrite oxidation were motivated primarily by the small change in bulk liquid DO, which could have supported higher NOB activity and caused loss of NOB suppression.

### Phase 4: Ammonium‐based aeration control (ABAC)

Phase 2 performance showed that a low bulk DO concentration could maintain a higher nitrite concentration and support anammox metabolism; however, nitrite was highly variable and made anammox‐based total N removal vulnerable to instability. The variability observed likely occurred because the available ammonium was periodically used up before the end of a cycle, which would have reduced AMX growth and supported NOB growth. Hence, ABAC was implemented during Phase 4 to maintain a minimum residual ammonium concentration throughout each reaction cycle to create a condition that suppressed NOB and supported AOB and AMX activity (Lotti et al., 2014; Pérez, Lotti, Kleerebezem, Picioreanu, & van Loosdrecht, 2014). For an SBR, the suppression of NOB by residual ammonium is much more pronounced at the beginning of the reaction phase than the end given the concentration gradient in the reactor. This also allows for sufficient free ammonia to be present that can inhibit NOB, despite the eventual decrease in total ammonia‐N by the end of each reaction cycle. The ABAC strategy used an ${\text{NH}}_{4}^{+}/{\text{NO}}_{3}^{-}$ sensor to maintain a residual ammonium concentration around 5 mg/L as N and a DO sensor to maintain a low bulk DO setpoint (0.5 mg O_2_/L). This strategy reduced variability in bulk liquid nutrient concentrations and increase aeration efficiency.

**Figure 5:**
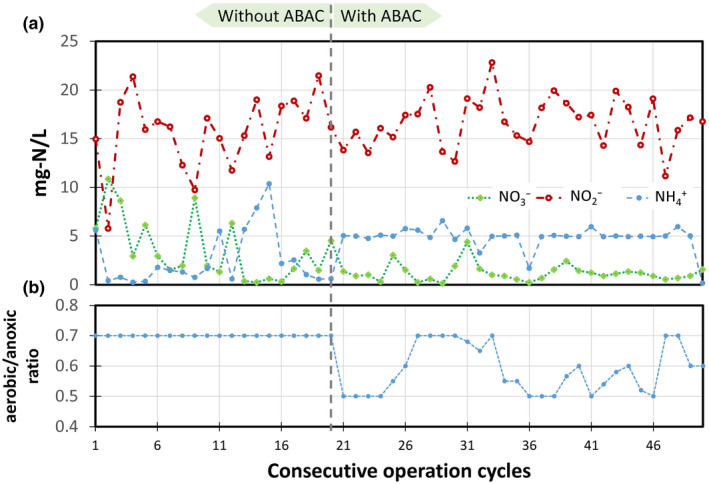
Reactor operation with and without ABAC (days from 396 to 417). (a) Effluent nitrogen species concentration profile under both scenarios (measurements determined using sensors and corrected with analytically determined values). (b) The corresponding total aerobic/anoxic duration fraction.

With ABAC, we saw a significant shift in both performance and microbial community population composition. Detectable effluent nitrite (16.0 ± 1.6 mg‐N/L) and nitrate (2.26 ± 0.72 mg‐N/L) resulted in a high NAR of 0.88 ± 0.04. Also, a lower residual ammonium concentration of 3.5 ± 1.3 mg/L was maintained. The average overall inorganic nitrogen removal efficiency improved slightly to 54.8 ± 3.3% compared to the other phases (Figure 5); however, the improvement was not statistically significant compared to both Phase 2 (*p* = 0.12) and Phase 3 (*p* = 0.75). Nevertheless, we saw evidence of improved anammox activity. From the in situ nitrification test on day 459 (Supporting Information Appendix S1: Table S8), AOB (0.31 g N‐NO_2_^−^ formed per g VSS per day) were 3.5 times more active than NOB (0.09 g N‐NO_3_^−^ formed per g VSS per day), while the in situ anammox activity test performed on day 463 resulted in an anammox specific activity of 0.153 mg‐N/mg‐VSS day, which is about 1.5 times faster than Phase 2 and almost 2 times faster than Phase 3 (see Supporting Information Appendix S1: Table S4). From the cross‐cycle data (Supporting Information Appendix S1: Tables S25 and S26) analysis, we estimated AMX contributed at least 40% of TIN removed. Illumina MiSeq analysis (day 460, Figure 3) provided additional evidence that the anammox population had recovered significantly during Phase 4 to a relative abundance of 13% while OHOs declined. Together, the performance and microbial community data demonstrate that coupling three aspects of SMC (low DO setpoint, intermittent aeration, and ABAC) created a favorable condition for partial nitritation/anammox in a granular system that received a simulated dilute mainstream anaerobic effluent and reduced the variability in TIN removal (Figure 4).

Our data also suggest that implementing ABAC improved aeration efficiency, as shown in Figure 5. To take a closer look at the improvement made by ABAC in our system, we show sensor‐based performance measurements from the reactor for 50 consecutive cycles as we transitioned from Phase 3 to Phase 4. ABAC determined the aerobic duration by limiting aeration up until the residual ammonium dropped below the setpoint of. This caused the overall aerobic duration to be shorter as compared to using a DO‐based setpoint only. In this case, the overall aerobic duration was reduced by up to 25% and on average by 15% with the use of ABAC versus DO setpoint operation with fixed aerobic duration (Figure 5b).

## Discussion

### Stable granulation is possible in a B‐stage nitrogen removing GSR system

Our results show that it is possible to produce stable granules in an aerobic granular sludge system receiving a dilute wastewater containing residual organic carbon and ammonium. Mature granules with a mean diameter of 1 mm were developed within two months of operation on a synthetic feed with a COD loading of 0.2 kg m^−3^ day^−1^ and nitrogen loading of 0.1 kg N m^−3^ day^−1^. The larger granules are likely to have a larger intra‐granular anaerobic zone that can support anaerobic metabolisms. Generally, granules are much easier to develop when the organic loading rate is higher than 1 kg m^−3^ day^−1^ (Jafari Kang & Yuan, 2017; Tay et al., 2004). A few studies have reported successful granulation at low organic loading rates (between 0.4 and 1 kg m^−3^ day^−1^) but with a longer time for stable granulation (65–120 days) than what was observed in this study (60 days) (Ni et al., 2009; Zhang et al., 2015). Therefore, with this study we demonstrated that it is possible to produce granules at low organic loading conditions pertinent to mainstream applications.

The organic carbon load to the GSR played two major roles. First, it was a major contributor for N removal in all phases. Using stoichiometry, we estimate that it functioned as an electron donor and contributed around 60%, 64%, and 60% of the oxidized TIN removal observed during phases 2, 3, and 4, respectively. The loss of organic carbon due to its reaction with oxidized TIN was limited to the first anoxic period and the availability of residual NO_X_. Any residual organic carbon present in the first aerobic zone was oxidized by O_2_. Second, the organic carbon load to the GSR provided the minimum loading needed to develop and sustain granules in our dilute system. Peyong, Zhou, Abdullah, and Vadivelu (2012) observed that reducing the organic loading rate below 0.54 kg COD m^−3^day^−1^ resulted in disintegration of granules and the subsequent loss of biomass. Hence, our study showed that it is indeed possible to maintain a stable granular system with organic loading as low as 0.2 kg COD m^−3^day^−1^.

### Low bulk DO with intermittent aeration supported NOB suppression

Operating the reactor at a bulk DO setpoint of 0.5 mg/L combined with intermittent aeration effectively suppressed NOB activity. The NAR observed during phases 2 and 4 was significantly higher than Phase 3 (*p* < 0.001) and indicates that operating at low bulk DO was a key to effective NOB suppression (Figure 4). Suppression of NOB by low DO is also known to occur in both activated sludge (Peng & Zhu, 2006) and biofilm systems (Brockmann & Morgenroth, 2010; Ma et al., 2015). Rapid intermittent aeration also suppressed NOB because they are known to adapt slowly under transient conditions when shifting from an anaerobic to an aerobic environment, and leads to an accumulated growth disadvantage (Gilbert et al., 2014; Kornaros et al., 2010; Regmi et al., 2014). Concurrently, specific to our system, the quick loss of both VFA and methane means the residual ammonia creates a condition that supports the growth of AOB and ANX more so than NOB for most of the reaction cycle. On top of this, the presence of residual ammonium between 2 and 5 mg NH_4_^−^ N/L has been reported to differentially limit the activity of NOB relative to that of AOB (Pérez et al., 2014; Poot, Hoekstra, Geleijnse, van Loosdrecht, & Pérez, 2016). Therefore, our SMC operation strategy with low and intermittent DO mainly favored AOB while suppressing NOB.

The success of using low bulk DO to achieve NOB suppression can be attributed in part to the known differences in growth rate between the AOB and NOB genera present in our system. A 16S rRNA gene‐based community analysis showed that the only NOB types detected in our reactor are from the genus *Nitrospira*, which are typically found to have a lower maximum specific growth rate and lower oxygen affinity (Blackburne, Vadivelu, Yuan, & Keller, 2007) than the AOB detected in our system, the genus *Nitrosomonas*, which are typically found to have a higher maximum specific growth rate and oxygen affinity (Blackburne, Yuan, & Keller, 2008). These two genera of bacteria have been found to coexist in many partial nitritation systems, as summarized by Cao et al. (2017) and reported by other authors (Sinha, Ajit, & Annachhatre, 2006; Wett et al., 2013). Hence, the AOB in our system are likely to have a higher oxygen affinity under low DO conditions than the NOB, resulting in suppression of the latter.

### Coupling ABAC with low DO setpoint enhanced energy‐efficient, anammox‐supported N removal

The performance of the GSR was stable, and aeration energy demand was reduced with the addition of ABAC. When the reactor was operated without residual ammonium control during Phase 3, it was not possible to consistently maintain residual ammonium through the end of the reaction cycle; hence, the residual ammonium concentration tended to vary substantially (Figure 5a). This is undesirable, since a minimum residual ammonium concentration is needed throughout the reaction zones for successful partial nitritation. Thus, this underscores the benefit brought by ABAC to ensure that an ammonium residue is maintained throughout the reaction time. When we added ABAC, aeration duration was reduced by up to 25% relative to what occurred when we used a DO setpoint only (Figure 5b). This reduction in aerobic duration translates into a reduction in aeration energy cost. Consequently, the use of ABAC resulted in tighter aeration control, which yielded more stable residual ammonium and overall TIN removal performance for the system. Translated to full‐scale treatment systems that often have a dynamic influent composition, these results imply that the use of ABAC will be critical to the cost‐effective deployment of mainstream B‐stage GSR applications that must achieve stable nitrogen reduction.

The use of ABAC with low DO setpoint and intermittent aeration also improved the retention of AMX in our system. Whole community (16S rRNA gene) sequencing data showed that coupling ABAC with low intermittent DO setpoint control corresponded with the recovery of AMX to around 13% relative abundance, four times higher than was seen without ABAC (Figure 3). The increase in AMX relative abundance corresponded with a 1.5‐fold increase in the in situ rate of anammox specific activity relative to what was observed during Phase 2 (low DO without ABAC). Furthermore, the specific anammox rate measured during Phase 4 (low DO with ABAC, 0.153 mg‐N/mg‐VSS‐day) is similar the rate reported by Lotti, Kleerebezem, and Loosdrecht (2015) for a partial nitritation/anammox SBR controlled with low DO and ABAC at 20°C (0.11 mg‐N/mg‐VSS day) and at 25°C (0.14 mg‐N/mg‐VSS day). To achieve N removal via nitrite by suppressing NOB, other studies have used DO, pH, and ORP in aerobic granular sludge reactors (Lochmatter, Gonzalez‐Gil, & Holliger, 2013; Tao, Gao, Fu, Wu, & Ren, 2012), while DO and ABAC have been used in conventional activated sludge systems (Regmi et al., 2015, 2014) to control DO setpoint and aerobic duration. All of these studies had higher organic and/or nitrogen volumetric loading than this study. Here, we developed and demonstrated a SMC strategy that integrated a DO setpoint, intermittent aeration, and residual ammonia control to promote partial nitritation/anammox in a mainstream GSR fed with dilute wastewater to achieve N removal with reduced aeration expense.

### Less aggressive start‐up is required for better nitrogen removal

The manner with which the GSR was operated during granulation influenced the system's ability to retain anammox activity. As the 16S rRNA sequencing results show, AMX was substantially reduced in abundance during the granulation period, consistent with the corresponding higher nitrite accumulation and lower levels of nitrate. We believe the observed reduction in AMX relative abundance has to do with two unfavorable start‐up conditions. First, during the start‐up period granules were developed with a short settling time to select against flocculent sludge. This created a short residence time of 4.8 ± 0.6 days (Supporting Information Appendix S1: Figure S1), which was less than the minimum reported doubling time of 11 days for AMX (Strous et al., 1998) in an SBR, and this possibly caused washout within the first few days before granules started developing. While others have predicted that SRTs as short as three days are possible (Lotti, Kleerebezem, Abelleira‐Pereira, Abbas, & Loosdrecht, 2015; Zhang et al., 2017), we did not observe that with our data. Second, since the influent contained only ammonium and not nitrite, AMX growth had to rely on AOB activity and achieving nitrite accumulation, neither of which was stable during the start‐up period.

Collectively, these results suggest that a less aggressive start‐up condition is required to retain a higher percentage AMX population. A less aggressive start‐up condition could be implemented to include (a) gradually decreasing the settling time to maintain an adequate SRT until granules start to appear; (b) supplementing the feed with nitrite during start‐up until the initial development of granules is observed; and (c) incorporating an anaerobic phase at the beginning of the run. The last two actions were demonstrated by Winkler et al. (2012) for integration of anammox to outcompete acetate in a granular system that received a higher organic (0.6 kg‐COD m^−3^ day^−1^) and N (1.14 kg‐N m^−3^ day^−1^) loading than this study. In addition to these actions, start‐up can be further improved by incorporating intermittent aeration to match the rate of nitritation with the rate of anammox activity in the system. This can be achieved by using a SMC to dynamically adjust the aerobic and anaerobic durations to promote partial nitritation/anammox while suppressing NOB. Further studies are needed to demonstrate the viability of these ideas.

## Conclusions

We demonstrated that successful N removal from a dilute mainstream wastewater requires a robust real‐time control strategy for effective utilization of resources and reduced energy expense. We showed that it is possible to develop granular sludge in a low carbon‐loaded system that can effectively suppress NOB activity so that N removal can be achieved via partial nitritation/anammox. Key operational strategies were identified and include using low DO intermittent aeration with ABAC (i.e., to maintain minimum residual ammonium). The findings of this research indicate that it is possible to remove nitrogen in a single compact system and with less aeration energy expended if simultaneous nitritation, anammox, and heterotrophic denitrification are enabled with the assistance of SMC.

## Conflict of interest

The authors declare no conflict of interest.
