# Supplementary material for: Sensor‐mediated granular sludge reactor for nitrogen removal and reduced aeration demand using a dilute wastewater
Source: Water Environ Res. 2020 Feb 5;92(7):1006–16. doi: 10.1002/wer.1296 (PMC7383604; doi:10.1002/wer.1296)
Supplement: Supplementary file 1 [file WER-92-1006-s001.docx]

**Supplementary Information for:**

**Sensor-Mediated Granular Sludge Reactor for Nitrogen Removal and Reduced Aeration Demand using a Dilute Wastewater**

1. **SRT calculation**

Solids are wasted from the reactor in each cycle only during the decanting period and sludge retention time (SRT) was calculated according to Eq. (S1). Figure SI-1 gives the box plot for the calculated SRT values for the four operation phases.

|  | $SRT=\frac{{TSS}_{r}\cdot V_{r}}{{TSS}_{eff}\cdot Q_{eff}}$ | (Eq. SI-1 ) |
| --- | --- | --- |

Where:

TSS_r_: TSS concentration in the reactor (gTSS L^-1^);

V_r_: reactor volume (L);

TSS_eff_.: TSS concentration in the effluent (gTSS L^-1^);

Q_eff._: effluent flow rate (L d^-1^);


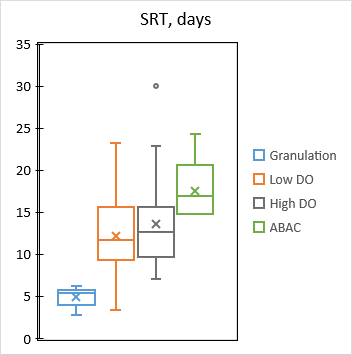


**Figure SI-1:** Box plot for SRT values during the four operation phases.

1. **Granule size distribution**

Granules were taken from the reactor, transferred to clear plate, and image was taken which was analyzed in ImageJ software for size distribution. Figure SI-2 gives granules size distributions for samples taken during Phase 1 and Phase 2.

| 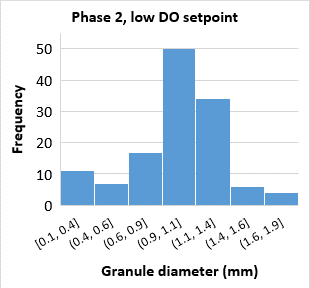 | 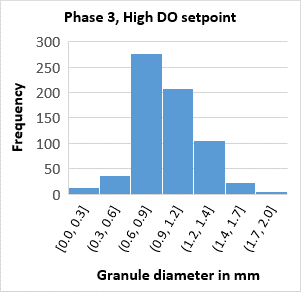 |
| --- | --- |

**Figure SI-2.** Granule size distribution from ImageJ software analysis.

1. **Solids concentration and SVI**

To determine biomass production and sludge volume index (SVI), total suspended solids (TSS), volatile suspended solids (VSS) and sludge volume were measured regularly. The sludge volume was measure by directly reading the settled granule depth from the reactor at 5 min and 30 min. The SVIs at 5 and 30 min were calculated by dividing the granule volume (mL) with reactor volume (4.5L) and TSS (g TSS L^-1^).

**Figure SI-2.** Solids concentration in the reactor and effluent.

**Figure SI-3.** Calculated SVI for 5 and 30 minutes.

1. **Long-term reactor’s performance data**

Figure SI4 gives the reactor’s N removal performance data from day 0 to day 474. In this figure ammonium concentration for the influent and effluent samples and all three N species (i.e., ammonium, nitrite, and nitrate) for effluent samples are shown. In addition, Table SI1 gives reactor’s data that are not included in the analysis due to known operational issues for completeness.


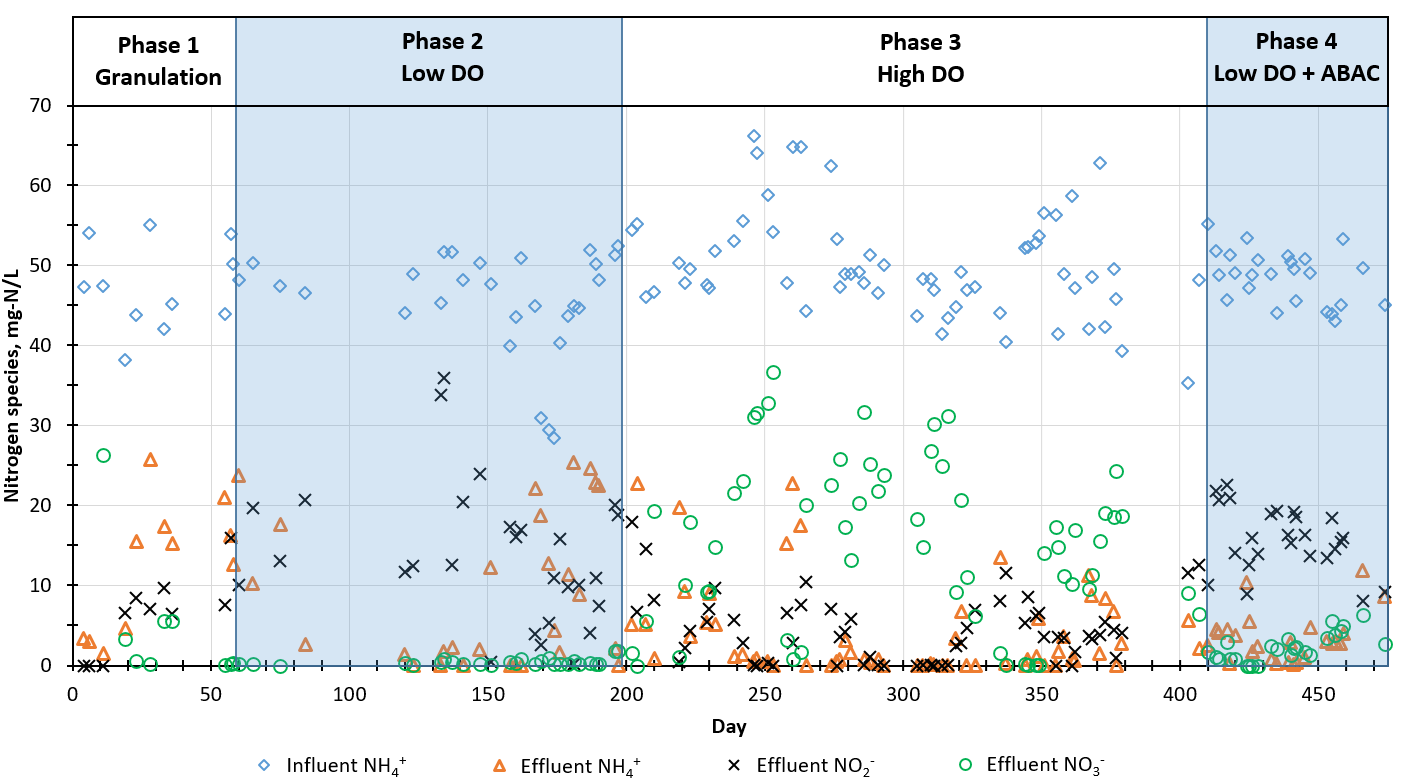


**Figure SI-4**. The reactor performance over 474 days of operation over four phases: Phase 1 granulation**;** Phase 2 low DO (~0.5 mg/L) setpoint**;** Phase 3 high DO (0.75 – 1 mg/L) setpoint**;** and Phase 4 ABAC with a DO setpoint of 0.5 mg/L. Samples for days when operational failures occurred are not shown but are summarized in Table SI-1. Water quality data shown based on analytical measurements and not sensor-based measurements.

**Table SI-1:** Summary of days the reactor has system failure

| **Day** | **Influent NH_4_^+^** | **Effluent NH_4_^+^** | **Effluent NO_2_^-^** | **Effluent NO_3_^-^** | **Problem** |
| --- | --- | --- | --- | --- | --- |
| 17 | 18.73 | 0.54 | 0.00 | 0.16 | Influent tubing leaking |
| 144 | 66.29 | 25.96 | 3.50 | 0.02 | Air tank ran out overnight |
| 200 | 51.79 | 28.17 | 4.10 | 0.00 | Diffusor stone clogged with biomass |
| 212 | 50.16 | 0.14 | 0.15 | 35.97 | Nitrogen gas run out |
| 215 | 48.07 | 28.20 | 1.02 | 2.56 | The DO was high probably air was leaking into the reactor |
| 256 | 46.61 | 28.62 | 2.88 | 1.74 | Very low DO in the reactor due air supply issue |
| 299 | 48.14 | 22.87 | 0.00 | 15.41 | Low Do in the reactor due to diffusor stone clogging |
| 301 | 45.96 | 0.00 | 0.02 | 39.53 | Too much air supply to the reactor |
| 383 | 48.05 | 0.00 | 0.00 | 38.11 | Problem with nitrogen gas supply |
| 390 | 48.47 | 28.23 | 0.07 | 0.00 | Air tank was empty |
| 391 | 43.52 | 27.75 | 1.09 | 0.65 | Reactor overflow due to decant pump failure |
| 395 | 51.27 | 22.03 | 1.24 | 27.00 | Nitrogen gas run out |
| 467 | 51.85 | 18.55 | 8.31 | 4.37 | Too low DO in the reactor |
| 468 | 54.99 | 24.04 | 3.04 | 3.68 | Error in ammonium sensor |

1. **Aeration control scheme**

The aeration control strategy developed uses a known intermittent aeration cycles with a DO setpoint and residual ammonium control (See Figure SI-5). Up to phase 3 only intermittent aeration with different DO setpoint (0.5 and 0.75 mg/L) was implement. This implies during these phases once the number of intermittent aeration and the durations of aerobic and anoxic cycles are fixed the total cycle duration is also fixed. For the last phase the residual ammonium control was used in addition to the intermittent aeration with a DO setpoint. This means, even if the number of intermittent cycles and the duration of aerobic and anoxic periods are fixed, the aerobic duration is variable as a result the total cycle duration becomes variable (i.e., it could be less than the predefined duration).


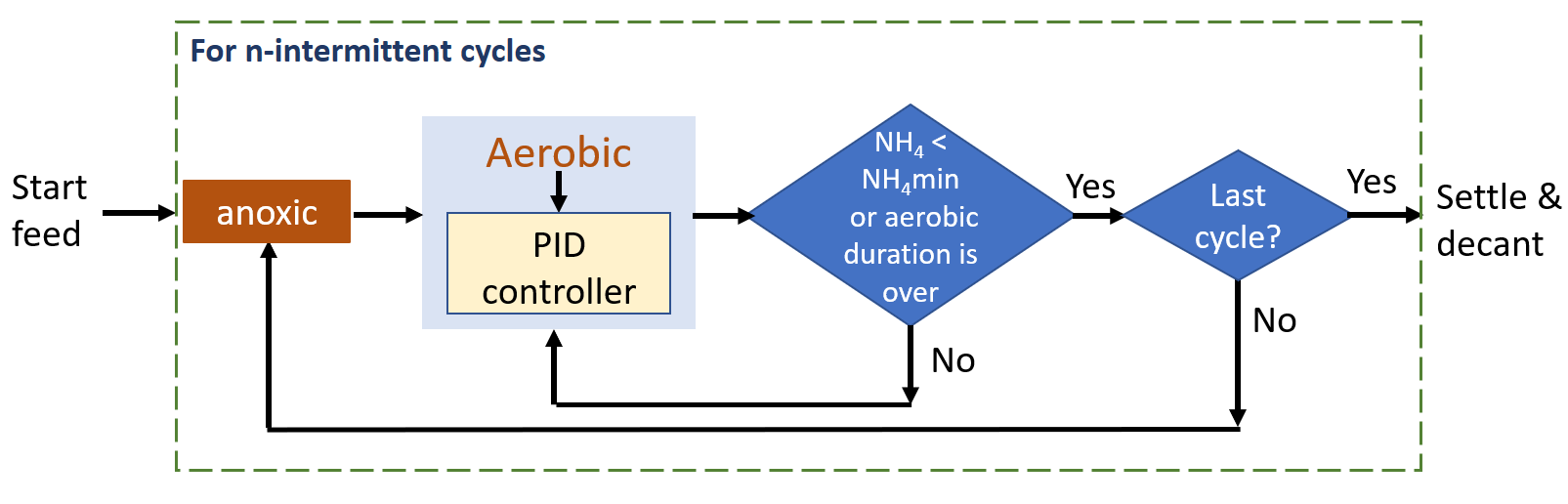


**Figure SI-5:** Aeration control design.

1. **VFA profile data**

A batch cycle done volatile fatty acids (VFAs) profile measurements were done using ion chromatography as described in Smith et al. (Smith et al., 2013). Data for samples taken during Phase 1 and Phase 2 operation period are given in Figure SI-6.

| A) | 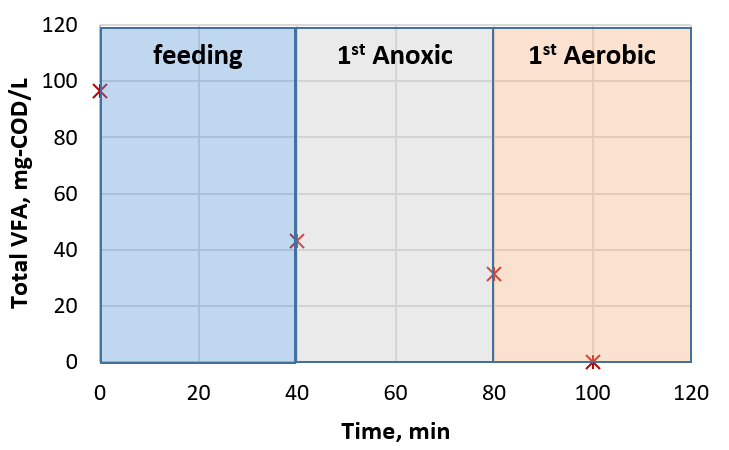 | B) | 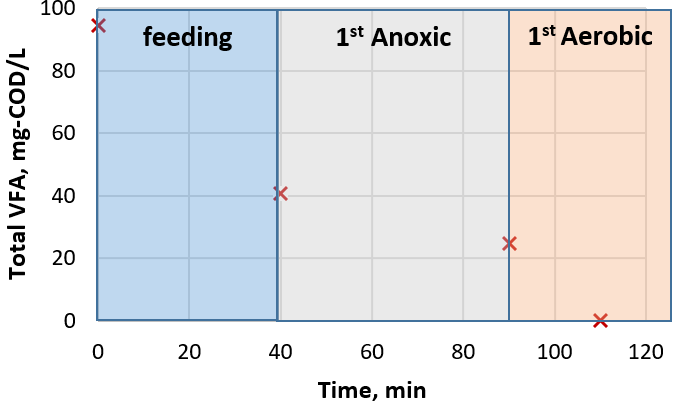 |
| --- | --- | --- | --- |

**Figure SI-6:** Total VFA profile during (a) Low DO day 127 and (b) High DO day 321.

1. **Reactor operation phases**

The different operation phases implemented for the entire operation period of the reactor are summarized in Table SI-2.

**Table SI-2.** Different phases of operation and aeration controls used.

|  | **Phase I**  **Granulation** | **Phase 2**  **Low DO control** | **Phase 3**  **High DO control** | **Phase 4**  **Low DO+Ammonium control** |
| --- | --- | --- | --- | --- |
| Operation days | 0-60 | 61-200 | 201-410 | 411-474 |
| DO setpoint (mg/L) | 1.5 | 0.5 | 0.75 | 0.5 |

1. **In-situ anammox and nitrification activities test**

The data for the in-situ anammox and nitrification test conducted for Phase 2 through Phase 4 are given from Table SI-3 to SI-7.

**Table SI-3:** In-situ anammox activity test during Phase 2 (low DO setpoint control) on day 155.

| **Time (min)** | **Nitrite** | | **Nitrate** | | **Ammonia** | |
| --- | --- | --- | --- | --- | --- | --- |
|  | mg-N/L | Error | mg-N/L | Error | mg-N/L | Error |
| 0 | 39.2 | 2.1 | 24.2 | 0.1 | 27.5 | 2.9 |
| 30 | 36.3 | 3.5 | 24.5 | 0.1 | 24.8 | 2.3 |
| 60 | 35.7 | 1.9 | 25.0 | 0.2 | 18.4 | 2.3 |
| 90 | 35.5 | 1.6 | 23.6 | 0.2 | 18.7 | 0.6 |
| 120 | 34.6 | 2.2 | 24.3 | 0.1 | 18.1 | 2.9 |
| 150 | 30.5 | 0.8 | 22.7 | 0.1 | 13.7 | 1.9 |
| 180 | 31.0 | 2.2 | 26.8 | 3.5 | 16.3 | 0.9 |
| 210 | 29.5 | 2.7 | 29.2 | 0.1 | 12.4 | 2.8 |
| 240 | 28.4 | 1.2 | 28.5 | 0.2 | 14.3 | 1.1 |

**Table SI-4:** In-situ anammox activity test during Phase 3, high DO setpoint control on day 362.

| **Time (min)** | **Nitrite** | | **Nitrate** | | **Ammonia** | |
| --- | --- | --- | --- | --- | --- | --- |
|  | mg-N/L | Error | mg-N/L | Error | mg-N/L | Error |
| Start fed | 0.0 | 0.0 | 0.0 | 0.0 | 27.4 | 1.0 |
| 0 | 20.2 | 0.3 | 8.1 | 0.0 | 25.4 | 0.8 |
| 30 | 20.5 | 0.1 | 8.2 | 0.0 | 22.6 | 0.5 |
| 45 | 19.5 | 0.2 | 8.5 | 0.5 | 22.0 | 0.3 |
| 60 | 19.0 | 0.1 | 8.8 | 0.5 | 21.5 | 1.1 |
| 75 | 19.5 | 0.1 | 8.8 | 0.0 | 20.6 | 1.1 |
| 90 | 19.0 | 0.1 | 9.0 | 0.0 | 20.8 | 0.7 |
| 105 | 17.4 | 0.1 | 9.1 | 0.0 | 20.4 | 0.5 |
| 120 | 17.2 | 0.2 | 9.6 | 0.0 | 19.3 | 0.8 |

**Table SI-5:** In-situ anammox activity test during Phase 4, low do setpoint plus ABAC control on day 463.

| **Time (min)** | **Nitrite** | | **Nitrate** | | **Ammonia** | |
| --- | --- | --- | --- | --- | --- | --- |
|  | mg-N/L | Error | mg-N/L | Error | mg-N/L | Error |
| Start fed | 28.4 | 0.7 | 0.0 | 0.0 | 30.0 | 1.0 |
| 0 | 23.5 | 0.7 | 24.2 | 0.1 | 25.3 | 0.4 |
| 30 | 18.2 | 0.5 | 24.5 | 0.1 | 20.5 | 1.5 |
| 60 | 15.2 | 0.2 | 25.0 | 0.2 | 18.2 | 1.0 |
| 90 | 13.2 | 0.3 | 25.5 | 0.2 | 15.2 | 0.7 |
| 120 | 12.1 | 0.5 | 26.0 | 0.1 | 14.0 | 1.3 |
| 150 | 11.3 | 0.5 | 26.7 | 0.1 | 13.2 | 2.1 |
| 180 | 10.5 | 0.4 | 26.8 | 3.5 | 12.2 | 1.0 |

**Table SI-6:** In-situ nitrification activity test during phase 2, low do setpoint control on day 179.

| **Time (min)** | **Nitrate** | | **Nitrite** | | **Ammonia** | |
| --- | --- | --- | --- | --- | --- | --- |
|  | mg-N/L | Error | mg-N/L | Error | mg-N/L | Error |
| 0 | 0.0 | 0.0 | 0.0 | 0.0 | 46.1 | 2.6 |
| 20 | 1.6 | 0.3 | 5.5 | 0.0 | 38.2 | 1.0 |
| 40 | 3.2 | 0.4 | 8.8 | 0.2 | 31.3 | 0.1 |
| 60 | 5.0 | 0.4 | 14.3 | 0.1 | 23.0 | 1.2 |
| 80 | 6.7 | 0.7 | 19.7 | 0.1 | 15.3 | 0.4 |
| 100 | 7.0 | 0.1 | 26.2 | 0.1 | 8.1 | 0.2 |
| 120 | 7.5 | 0.3 | 32.0 | 0.1 | 1.7 | 0.3 |

**Table SI-7:** In-situ nitrification activity test during Phase 3, high do setpoint control on day 376.

| **Time (min)** | **Nitrate** | | **Nitrite** | | **Ammonia** | |
| --- | --- | --- | --- | --- | --- | --- |
|  | mg-N/L | Error | mg-N/L | Error | mg-N/L | Error |
| 0 | 0.0 | 0.0 | 0.0 | 0.0 | 46.1 | 2.6 |
| 20 | 5.5 | 0.0 | 2.4 | 0.1 | 38.2 | 1.0 |
| 40 | 9.8 | 0.2 | 5.1 | 0.7 | 31.3 | 0.1 |
| 60 | 13.9 | 0.0 | 7.4 | 0.5 | 23.0 | 1.2 |
| 80 | 18.9 | 0.0 | 10.4 | 0.8 | 15.3 | 0.4 |
| 100 | 24.9 | 0.2 | 9.1 | 0.5 | 8.1 | 0.2 |
| 120 | 30.1 | 0.3 | 10.1 | 0.4 | 1.7 | 0.3 |

**Table SI-8**: In-situ nitrification activity test during Phase 4, low DO and ABAC on day 459

| **Time (min)** | **Nitrate** | | **Nitrite** | | **Ammonia** | |
| --- | --- | --- | --- | --- | --- | --- |
|  | mg-N/L | Error | mg-N/L | Error | mg-N/L | Error |
| 0 | 4.6 | 0.1 | 5.8 | 0.0 | 51.7 | 0.4 |
| 20 | 10.5 | 0.2 | 9.3 | 0.0 | 41.7 | 0.5 |
| 40 | 14.5 | 0.2 | 11.3 | 0.0 | 33.5 | 1.0 |
| 60 | 19.5 | 0.1 | 14.1 | 0.1 | 25.5 | 0.5 |
| 80 | 23.7 | 0.1 | 16.0 | 0.0 | 18.6 | 1.1 |
| 100 | 26.2 | 0.1 | 17.5 | 0.1 | 13.7 | 0.4 |
| 120 | 28.6 | 0.1 | 18.2 | 0.1 | 10.5 | 0.7 |
| 140 | 35.7 | 0.9 | 19.5 | 0.2 | 3.4 | 0.5 |

1. **Methane data**

Dissolved methane samples were directly taken from the reactor and analyzed in gas chromatography. Figure SI-7 shows methane profile for sample taken on day 420 during phase 4 of the reactor operation.
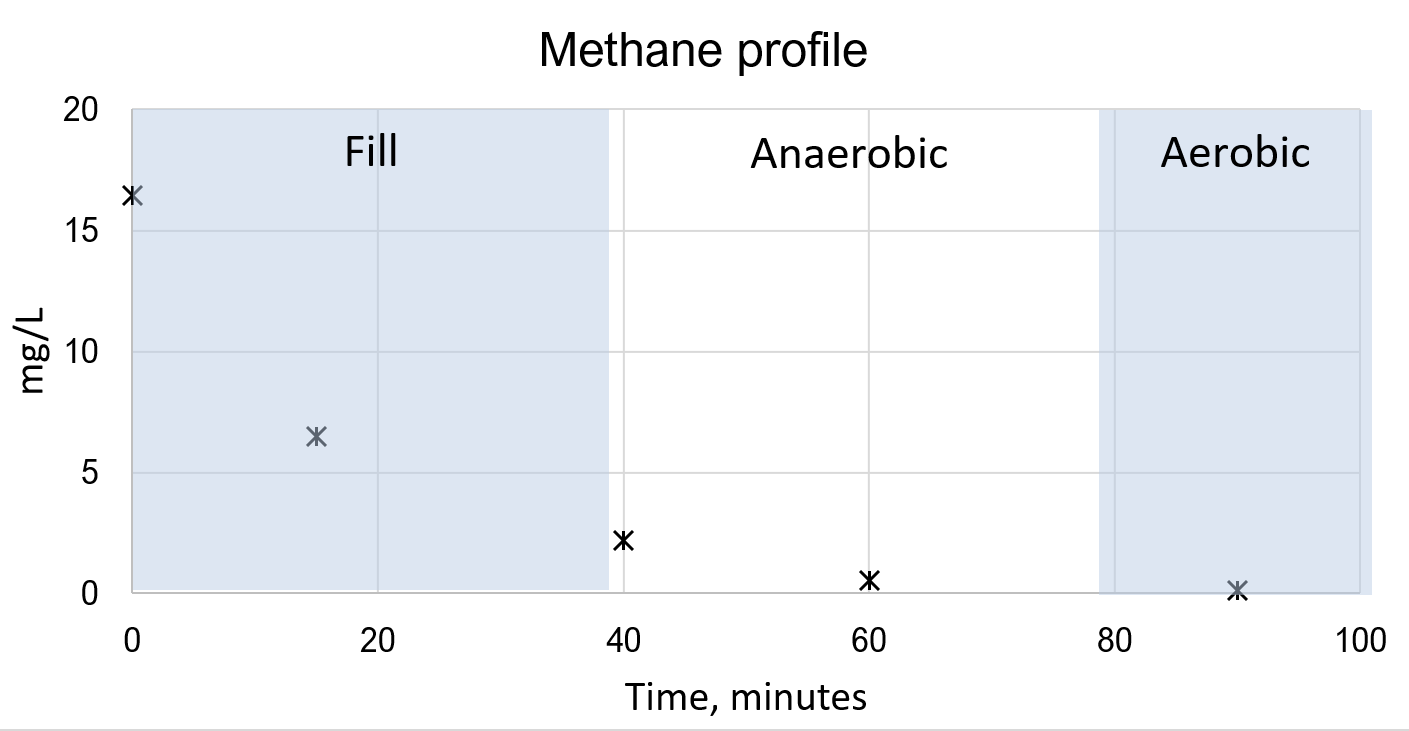


**Figure SI-7.** Methane profile taken on day 420 during Phase 4.

1. **Profile (Cross-cycle) data analysis**

Routine time profile (cross-cycle) monitoring of N species was done by taking sample at the different stages within one batch cycle. Tables SI-9 through SI-26 give the data for samples taken during the different operation stages of the reactor. These data were used to estimate the relative contribution of anammox vs OHOs for N removal. From the VFA data we noticed that by the end of the first aerobic period all VFA in the fed was consumed, hence we assumed N removal that has happened starting the second anoxic period is due to anammox plus N used for maintenance. With this assumption we used the following standard stoichiometric equations to determine the contribution of OHOs in N removal from fed to the end of the first anoxic period based on how much NO_X_ was lost for each cross-cycle data. We calculated OHOs contribution for a range of VFA fractions from 0 to 1 consumed for denitrification, while the remaining fraction is assumed to be oxidized in first aerobic period. Among the range of values, the maximum stoichiometrically possible VFA value was used to report the contribution of OHOs in N removal. This is done not to overestimate anammox contribution in N removal.

| NO_3_^-^ as electron acceptor | $0.125CH_{2}COO^{-}+0.106NO_{3}^{-}+0.0235NH_{4}^{+}+0.106H^{+}=0.155H_{2}O+0.031CO_{2}+0.053N_{2}+0.0235C_{5}H_{7}O_{2}N$ | (Eq. SI-2 ) |
| --- | --- | --- |
| NO_2_^-^ as electron acceptor | $0.125CH_{2}COO^{-}+0.177NO_{2}^{-}+0.0235NH_{4}^{+}+0.177H^{+}=0.1898H_{2}O+0.031CO_{2}+0.088N_{2}+0.0235C_{5}H_{7}O_{2}N$ | (Eq. SI-3 ) |

**Table SI-9:** Cross-cycle samples data for phase 2 taken on day 78

| **Time from start (min)** | **Stage** | **NH_4_^+^ (mg∙N/L)** | **NO_2_^-^ (mg∙N/L)** | **NO_3_^-^ (mg∙N/L)** |
| --- | --- | --- | --- | --- |
|  | Influent | 48.27 | 0 | 0 |
| 0 | Anoxic 11 | 25.66 | 9.85 | 0.64 |
| 50 | Anoxic 12 | 22.89 | 6.31 | 0.67 |
| 140 | Aerobic 12 | 17.77 | 9.28 | 4.20 |
| 190 | Anoxic 22 | 16.83 | 9.94 | 3.91 |
| 280 | Aerobic 22 | 4.91 | 18.10 | 2.16 |
| 285 | Effluent | 3.05 | 19.71 | 1.28 |

**Table SI-10:** Cross-cycle samples data for phase 2 taken on day 89

| **Time from start (min)** | **Stage** | **NH_4_^+^ (mg∙N/L)** | **NO_2_^-^ (mg∙N/L)** | **NO_3_^-^ (mg∙N/L)** |
| --- | --- | --- | --- | --- |
|  | Influent | 50.11 | 0.00 | 0.00 |
| 0 | Anoxic 11 | 32.94 | 4.61 | 0.18 |
| 50 | Anoxic 12 | 32.10 | 3.75 | 0.14 |
| 140 | Aerobic 12 | 28.55 | 6.18 | 0.17 |
| 190 | Anoxic 22 | 23.98 | 6.14 | 0.15 |
| 280 | Aerobic 22 | 20.55 | 9.66 | 0.21 |
| 285 | Effluent | 15.76 | 11.21 | 0.35 |

**Table SI-11:** Cross-cycle samples data for phase 2 taken on day 109

| **Time from start (min)** | **Stage** | **NH_4_^+^ (mg∙N/L)** | **NO_2_^-^ (mg∙N/L)** | **NO_3_^-^ (mg∙N/L)** |
| --- | --- | --- | --- | --- |
|  | Influent | 51.04 | 0.00 | 0.00 |
| 0 | Anoxic 11 | 29.31 | 3.12 | 1.22 |
| 50 | Anoxic 12 | 24.44 | 1.63 | 1.19 |
| 140 | Aerobic 12 | 17.21 | 2.67 | 1.29 |
| 190 | Anoxic 22 | 13.56 | 3.12 | 1.34 |
| 280 | Aerobic 22 | 8.50 | 5.64 | 1.65 |
| 285 | Effluent | 7.58 | 6.06 | 2.44 |

**Table SI-12:** Cross-cycle samples data for phase 2 taken on day 120

| **Time from start (min)** | **Stage** | **NH_4_^+^ (mg∙N/L)** | **NO_2_^-^ (mg∙N/L)** | **NO_3_^-^ (mg∙N/L)** |
| --- | --- | --- | --- | --- |
|  | Influent | 51.04 | 0.00 | 0.00 |
| 0 | Anoxic 11 | 26.24 | 8.30 | 0.17 |
| 50 | Anoxic 12 | 23.53 | 1.01 | 0.03 |
| 140 | Aerobic 12 | 20.30 | 11.89 | 0.13 |
| 190 | Anoxic 22 | 18.52 | 18.70 | 0.13 |
| 280 | Aerobic 22 | 10.23 | 19.21 | 0.39 |
| 285 | Effluent | 8.51 | 20.52 | 0.33 |

**Table SI-13:** Cross-cycle samples data for phase 2 taken on day 134

| **Time from start (min)** | **Stage** | **NH_4_^+^ (mg∙N/L)** | **NO_2_^-^ (mg∙N/L)** | **NO_3_^-^ (mg∙N/L)** |
| --- | --- | --- | --- | --- |
|  | Influent | 51.04 | 0.00 | 0.00 |
| 0 | Anoxic 11 | 33.02 | 17.95 | 0.17 |
| 50 | Anoxic 12 | 18.12 | 18.80 | 0.41 |
| 140 | Aerobic 12 | 8.13 | 30.76 | 0.27 |
| 190 | Anoxic 22 | 3.36 | 36.14 | 0.24 |
| 280 | Aerobic 22 | 0.10 | 38.66 | 0.26 |
| 285 | Effluent | 15.00 | 35.90 | 0.33 |

**Table SI-14:** Cross-cycle samples data for phase 2 taken on day 148

| **Time from start (min)** | **Stage** | **NH_4_^+^ (mg∙N/L)** | **NO_2_^-^ (mg∙N/L)** | **NO_3_^-^ (mg∙N/L)** |
| --- | --- | --- | --- | --- |
|  | Influent | 51.04 | 0.00 | 0.00 |
| 0 | Anoxic 11 | 33.02 | 4.02 | 0.17 |
| 50 | Anoxic 12 | 24.44 | 1.81 | 1.19 |
| 140 | Aerobic 12 | 17.21 | 6.14 | 1.29 |
| 190 | Anoxic 22 | 13.56 | 5.12 | 1.20 |
| 280 | Aerobic 22 | 8.50 | 7.85 | 0.95 |
| 285 | Effluent | 7.58 | 8.52 | 0.33 |

**Table SI-15:** Cross-cycle samples data for phase 2 taken on day 159

| **Time from start (min)** | **Stage** | **NH_4_^+^ (mg∙N/L)** | **NO_2_^-^ (mg∙N/L)** | **NO_3_^-^ (mg∙N/L)** |
| --- | --- | --- | --- | --- |
|  | Influent | 50.14 | 0 | 0 |
| 0 | Anoxic 11 | 31.41 | 5.01 | 1.22 |
| 50 | Anoxic 12 | 27.90 | 8.61 | 1.19 |
| 140 | Aerobic 12 | 20.08 | 15.21 | 1.29 |
| 190 | Anoxic 22 | 19.27 | 14.98 | 1.2 |
| 280 | Aerobic 22 | 12.67 | 16.68 | 0.95 |
| 285 | Effluent | 12.67 | 17.32 | 2.44 |

**Table SI-16:** Cross-cycle samples data for phase 2 taken on day 171

| **Time from start (min)** | **Stage** | **NH_4_^+^ (mg∙N/L)** | **NO_2_^-^ (mg∙N/L)** | **NO_3_^-^ (mg∙N/L)** |
| --- | --- | --- | --- | --- |
|  | Influent | 45.22 | 0 | 0 |
| 0 | Anoxic 11 | 30.22 | 5.845 | 0.24 |
| 50 | Anoxic 12 | 29.57 | 3.98 | 0.15 |
| 140 | Aerobic 12 | 23.53 | 6.71 | 0.19 |
| 190 | Anoxic 22 | 21.18 | 8.28 | 0.15 |
| 280 | Aerobic 22 | 14.55 | 12.99 | 0.33 |
| 285 | Effluent | 15.22 | 13.69 | 0.48 |

**Table SI-17:** Cross-cycle samples data for phase 3 taken on day 201

| **Time from start (min)** | **Stage** | **NH_4_^+^ (mg∙N/L)** | **NO_2_^-^ (mg∙N/L)** | **NO_3_^-^ (mg∙N/L)** |
| --- | --- | --- | --- | --- |
|  | Influent | 52.83 | 0.00 | 0.00 |
| 0 | Anoxic 00 | 28.13 | 8.81 | 0.07 |
| 25 | Anoxic 11 | 15.68 | 8.63 | 0.16 |
| 50 | Anoxic 12 | 29.58 | 3.99 | 0.13 |
| 75 | Aerobic 11 | 27.83 | 2.12 | 0.12 |
| 100 | Aerobic 12 | 26.21 | 2.13 | 0.19 |
| 150 | Anoxic 22 | 24.40 | 1.91 | 0.16 |
| 175 | Aerobic 21 | 22.78 | 4.81 | 0.13 |
| 200 | Aerobic 22 | 20.94 | 5.29 | 0.05 |
| 250 | Anoxic 32 | 17.35 | 7.75 | 0.16 |
| 275 | Aerobic 31 | 14.09 | 9.42 | 0.13 |
| 300 | Aerobic 32 | 12.10 | 10.72 | 0.35 |
| 205 | Effluent | 13.44 | 12.61 | 0.13 |

**Table SI-18:** Cross-cycle samples data for phase 3 taken on day 219

| **Time from start (min)** | **Stage** | **NH_4_^+^ (mg∙N/L)** | **NO_2_^-^ (mg∙N/L)** | **NO_3_^-^ (mg∙N/L)** |
| --- | --- | --- | --- | --- |
|  | Influent | 55.17 | 0.00 | 0.00 |
| 0 | Anoxic 00 | 38.96 | 3.71 | 0.07 |
| 25 | Anoxic 11 | 29.24 | 4.78 | 0.16 |
| 50 | Anoxic 12 | 35.09 | 1.09 | 0.13 |
| 75 | Aerobic 11 | 32.44 | 1.26 | 0.12 |
| 100 | Aerobic 12 | 29.21 | 1.73 | 0.19 |
| 150 | Anoxic 22 | 26.78 | 3.33 | 0.16 |
| 175 | Aerobic 21 | 23.50 | 4.26 | 0.13 |
| 200 | Aerobic 22 | 20.00 | 6.05 | 0.05 |
| 250 | Anoxic 32 | 20.42 | 8.96 | 0.16 |
| 275 | Aerobic 31 | 16.34 | 11.67 | 0.13 |
| 300 | Aerobic 32 | 13.64 | 10.68 | 0.35 |
| 205 | Effluent | 22.75 | 7.41 | 0.13 |

**Table SI-19:** Cross-cycle samples data for phase 3 taken on day 239

| **Time from start (min)** | **Stage** | **NH_4_^+^ (mg∙N/L)** | **NO_2_^-^ (mg∙N/L)** | **NO_3_^-^ (mg∙N/L)** |
| --- | --- | --- | --- | --- |
|  | Influent | 53.06 | 0.00 | 0.00 |
| 0 | Anoxic 00 | 27.09 | 2.85 | 10.80 |
| 25 | Anoxic 11 | 23.21 | 2.56 | 12.51 |
| 50 | Anoxic 12 | 21.60 | 1.29 | 12.74 |
| 75 | Aerobic 11 | 17.97 | 2.97 | 13.28 |
| 100 | Aerobic 12 | 14.83 | 4.30 | 15.12 |
| 150 | Anoxic 22 | 12.96 | 4.29 | 15.66 |
| 175 | Aerobic 21 | 9.68 | 4.75 | 16.41 |
| 200 | Aerobic 22 | 7.41 | 5.93 | 17.89 |
| 250 | Anoxic 32 | 6.19 | 5.66 | 18.47 |
| 275 | Aerobic 31 | 5.15 | 5.73 | 18.77 |
| 300 | Aerobic 32 | 4.00 | 5.57 | 19.97 |
| 205 | Effluent | 1.12 | 5.70 | 21.61 |

**Table SI-20:** Cross-cycle samples data for phase 3 taken on day 246

| **Time from start (min)** | **Stage** | **NH_4_^+^ (mg∙N/L)** | **NO_2_^-^ (mg∙N/L)** | **NO_3_^-^ (mg∙N/L)** |
| --- | --- | --- | --- | --- |
|  | Influent | 53.06 | 0.00 | 0.00 |
| 0 | Anoxic 00 | 26.77 | 0.02 | 15.53 |
| 25 | Anoxic 11 | 9.70 | 0.00 | 23.59 |
| 50 | Anoxic 12 | 25.29 | 0.00 | 11.57 |
| 75 | Aerobic 11 | 21.84 | 1.94 | 12.55 |
| 100 | Aerobic 12 | 19.10 | 2.23 | 13.79 |
| 150 | Anoxic 22 | 15.37 | 2.19 | 15.78 |
| 175 | Aerobic 21 |  |  |  |
| 200 | Aerobic 22 |  |  |  |
| 250 | Anoxic 32 | 5.27 | 3.26 | 23.70 |
| 275 | Aerobic 31 | 3.82 | 2.44 | 24.84 |
| 300 | Aerobic 32 | 0.69 | 0.57 | 29.62 |
| 205 | Effluent | 0.48 | 0.47 | 31.05 |

**Table SI-21:** Cross-cycle samples data for phase 3 taken on day 253

| **Time from start (min)** | **Stage** | **NH_4_^+^ (mg∙N/L)** | **NO_2_^-^ (mg∙N/L)** | **NO_3_^-^ (mg∙N/L)** |
| --- | --- | --- | --- | --- |
|  | Influent | 54.16 | 0.00 | 0.00 |
| 0 | Anoxic 00 | 27.08 | 0.00 | 16.82 |
| 25 | Anoxic 11 | 17.19 | 0.00 | 19.81 |
| 50 | Anoxic 12 | 17.07 | 0.00 | 18.33 |
| 75 | Aerobic 11 | 14.58 | 1.41 | 19.26 |
| 100 | Aerobic 12 | 9.20 | 2.49 | 22.89 |
| 150 | Anoxic 22 | 6.72 | 2.73 | 25.26 |
| 175 | Aerobic 21 | 2.84 | 3.03 | 29.58 |
| 200 | Aerobic 22 | 0.94 | 0.75 | 34.35 |
| 250 | Anoxic 32 | 0.28 | 0.00 | 35.86 |
| 275 | Aerobic 31 | 0.21 | 0.00 | 36.11 |
| 300 | Aerobic 32 | 0.02 | 0.00 | 36.87 |
| 205 | Effluent | 0.01 | 0.00 | 36.64 |

**Table SI-22:** Cross-cycle samples data for phase 3 taken on day 274

| **Time from start (min)** | **Stage** | **NH_4_^+^ (mg∙N/L)** | **NO_2_^-^ (mg∙N/L)** | **NO_3_^-^ (mg∙N/L)** |
| --- | --- | --- | --- | --- |
|  | Influent | 62.45 | 0.00 | 0.00 |
| 0 | Anoxic 00 | 31.24 | 3.55 | 13.28 |
| 25 | Anoxic 11 | 25.29 | 5.60 | 9.45 |
| 50 | Anoxic 12 | 25.24 | 5.78 | 4.88 |
| 75 | Aerobic 11 | 21.38 | 7.26 | 6.25 |
| 100 | Aerobic 12 | 14.65 | 9.73 | 9.93 |
| 150 | Anoxic 22 | 13.46 | 9.79 | 10.74 |
| 175 | Aerobic 21 | 7.08 | 6.66 | 13.97 |
| 200 | Aerobic 22 | 3.74 | 10.26 | 17.01 |
| 250 | Anoxic 32 | 2.58 | 9.05 | 17.09 |
| 275 | Aerobic 31 | 0.51 | 8.89 | 19.32 |
| 300 | Aerobic 32 | 1.07 | 7.55 | 21.59 |
| 205 | Effluent | 0.04 | 7.11 | 22.56 |

**Table SI-23:** Cross-cycle samples data for phase 3 taken on day 284

| **Time from start (min)** | **Stage** | **NH_4_^+^ (mg∙N/L)** | **NO_2_^-^ (mg∙N/L)** | **NO_3_^-^ (mg∙N/L)** |
| --- | --- | --- | --- | --- |
|  | Influent | 49.25 | 0.00 | 0.00 |
| 0 | Anoxic 00 | 25.43 | 5.00 | 9.50 |
| 25 | Anoxic 11 | 42.84 | 0.00 | 1.03 |
| 50 | Anoxic 12 | 35.42 | 1.06 | 0.63 |
| 75 | Aerobic 11 |  |  |  |
| 100 | Aerobic 12 | 33.83 | 3.10 | 2.97 |
| 150 | Anoxic 22 | 22.67 | 7.98 | 7.33 |
| 175 | Aerobic 21 |  |  |  |
| 200 | Aerobic 22 | 13.83 | 11.36 | 12.91 |
| 250 | Anoxic 32 | 9.72 | 12.49 | 15.10 |
| 275 | Aerobic 31 |  |  |  |
| 300 | Aerobic 32 | 3.32 | 10.39 | 19.75 |
| 205 | Effluent | 3.12 | 9.98 | 20.36 |

**Table SI-24:** Cross-cycle samples data for phase 3 taken on day 326

| **Time from start (min)** | **Stage** | **NH_4_^+^ (mg∙N/L)** | **NO_2_^-^ (mg∙N/L)** | **NO_3_^-^ (mg∙N/L)** |
| --- | --- | --- | --- | --- |
|  | Influent | 47.29 | 0.00 | 0.00 |
| 0 | Anoxic 00 | 23.68 | 2.93 | 0.76 |
| 25 | Anoxic 11 | 22.61 | 0.14 | 0.00 |
| 50 | Anoxic 12 | 22.39 | 0.00 | 0.00 |
| 75 | Aerobic 11 |  |  |  |
| 100 | Aerobic 12 | 17.71 | 3.30 | 0.70 |
| 150 | Anoxic 22 | 17.08 | 3.17 | 0.86 |
| 175 | Aerobic 21 |  |  |  |
| 200 | Aerobic 22 | 7.21 | 7.26 | 2.69 |
| 250 | Anoxic 32 | 3.06 | 8.36 | 3.45 |
| 275 | Aerobic 31 |  |  |  |
| 300 | Aerobic 32 | 0.09 | 8.90 | 6.18 |
| 205 | Effluent | 0.06 | 6.92 | 6.23 |

**Table SI-25:** Cross-cycle samples data for phase 4 taken on day 434

| **Time from start (min)** | **Stage** | **NH_4_^+^ (mg∙N/L)** | **NO_2_^-^ (mg∙N/L)** | **NO_3_^-^ (mg∙N/L)** |
| --- | --- | --- | --- | --- |
|  | Influent | 44.02 | 0.00 | 0.00 |
| 0 | Anoxic 00 | 15.97 | 5.90 | 0.41 |
| 30 | Anoxic 11 | 15.15 | 3.73 | 0.12 |
| 60 | Anoxic 12 | 12.58 | 0.95 | 0.47 |
| 90 | Aerobic 1 | 10.88 | 3.94 | 0.68 |
| 120 | Anoxic 21 | 8.19 | 3.57 | 0.71 |
| 150 | Anoxic 22 | 7.97 | 2.37 | 1.12 |
| 180 | Aerobic 2 | 6.38 | 4.36 | 1.28 |
| 210 | Anoxic 31 | 3.10 | 4.07 | 1.51 |
| 240 | Anoxic 32 | 2.49 | 2.68 | 3.99 |

**Table SI-26:** Cross-cycle samples data for phase 4 taken on day 447

| **Time from start (min)** | **Stage** | **NH_4_^+^ (mg∙N/L)** | **NO_2_^-^ (mg∙N/L)** | **NO_3_^-^ (mg∙N/L)** |
| --- | --- | --- | --- | --- |
|  | Influent | 44.02 | 0.00 | 0.00 |
| 0 | Anoxic 0 | 22.67 | 9.47 | 0.00 |
| 40 | Anoxic 1 | 21.33 | 9.33 | 0.29 |
| 70 | Aerobic 11 | 17.06 | 13.05 | 0.44 |
| 100 | Aerobic 12 | 13.05 | 13.56 | 0.58 |
| 140 | Anoxic 2 | 12.79 | 14.53 | 0.89 |
| 170 | Aerobic 21 | 8.98 | 15.30 | 0.72 |
| 200 | Aerobic 22 | 6.67 | 18.04 | 0.84 |
| 240 | Anoxic 3 | 6.52 | 19.54 | 1.07 |
| 270 | Aerobic 31 | 3.79 | 21.59 | 1.24 |
| 300 | Aerobic 32 | 3.33 | 21.26 | 1.15 |

1. **Physical characterization of granules**

To determine biomass production and sludge volume index (SVI), total suspended solids (TSS), volatile suspended solids (VSS) and sludge volume were measured weekly or biweekly. The sludge volume was measure by directly reading the settled granule depth from the reactor at 5 min and 30 min. The SVIs at 5 and 30 min were calculated by dividing the granule volume (mL) with reactor volume (4.5L) and TSS (g TSS L-1). The morphology of the granules was monitored periodically using a digital camera (Canon G7X) and ImageJ software (Rasband and S., 2012) for image analysis of the granules’ size distribution. Solids were wasted at the end of each cycle during the decanting period only and was considered when calculating the solids retention time (SRT) as described in the Section 1.

1. **Synthetic media**

The influent fed to the reactor was a synthetic anaerobic effluent freshly prepared every 2-days. Twenty liters of influent was prepared every 2-3 days by combining an acidic trace metals, basic trace metals and NaEDTA (10.08g/L) each 20 mL. The acidic trace metals stock solution was prepared by mixing (per liter): CoCl_2_·6H_2_O, 0.28 g; ZnSO_4_·7H_2_O, 0.34 g; H_3_BO_3_, 37 mg; MnCl_2_·4H_2_O, 0.11 g; AlCl_3_·6H_2_O, 28 mg; NiCl_2_·6H_2_O, 0.14 g; CuCl_2_·2H_2_O, 0.10 g. The basic trace metals stock solution was prepared by mixing (per liter): (NH_4_)_2_MoO_4_·4H_2_O, 0.16 g; Na_2_SeO_4_, 22 mg; Na_2_WO_4_·2H_2_O, 39.5 mg. Next, 100 mL of a 56g/L ammonium bicarbonate stock was added. After thoroughly mixing these in a flask, 20 mL of a chloride salts solution made up of (per liter): CaCl_2_·2H_2_O, 5g; MgCl_2_·6H_2_O, 33 g; KCl, 16 g; KH_2_PO_4_, 11 g, was added to the mixture. This mixture was combined with 65 mL of 1 N HCL and diluted with DI water to make 20 liters of influent solution. Following this, the solution was sparged with methane gas for 25 minutes, after which 95 mL of a 30 g/L sodium bicarbonate stock and 20 mL of a 6.6 g/L FeSO_4_·7H_2_O stock (stored in an anaerobic glove chamber) were added. Finally, the influent solution was sparged for 5 more minutes with methane gas. For the addition of VFAs, a separate bottle containing equivalent COD proportions of sodium acetate and sodium propionate was fed via a peristaltic pump to get 100 mg COD/L. All glassware used for preparing the feed stock was autoclaved every time before making influent.

1. **In-situ activity batch tests**

In-situ batch activity tests were conducted to determine the extent of nitrification and anaerobic ammonium oxidation (anammox). For these in-situ tests the reactor was operated for at least one hour under aerobic conditions at the end of the prior cycle to remove any remaining organic carbon present in the system. Next, the supernatant was decanted completely, granules were taken out of the reactor, and rinsed with VFA- and methane-free influent media. The washed granules were returned to the reactor, which was filled with VFA- and methane-free influent media containing inorganic nitrogen chemicals tailored for each test. Specifically, ammonium was the only inorganic nitrogen chemical added for the in-situ nitrification test, while ammonium, nitrite and nitrate (to create an appropriate redox condition and avoid sulfate reduction (Loosdrecht et al., 2016)) were added for the in-situ anammox activity test. For the in-situ nitrification test, the reactor was operated under aerobic conditions by mixing with dry air gas and taking samples after a stable bulk DO > 1 mg/L was achieved. For the in-situ anammox test, the reactor was operated anaerobically by mixing with nitrogen gas (99.998% purity), and taking samples after achieving stable anaerobic conditions, defined by waiting 10 minutes after no DO was detected (detection limit = 0.00 mg/L O_2_). Seven to nine samples were taken over 2 to 4 hours for each test and analyzed for ammonium, nitrite and nitrate according to the methods described in Section 2.5. One of each kind of test occurred during each of Phase 2, 3 and 4. The maximum specific anammox rate (mg-N/mg-VSS/day) was determined by first doing linear regression using the data to determine the nitrogen removal rate (i.e., ammonium removal rate plus nitrite removal rate minus nitrate production rate) then dividing it by the volatile suspended solids.

**References**

Loosdrecht, M.C.M. van, Nielsen, P.H., Lopez-Vazquez, C.M., Brdjanovic, D., 2016. Experimental procedures in wastewater treatment, Experimental procedures in wastewater treatment. IWA Publishing.

Rasband, W.S., S., W., 2012. ImageJ: Image processing and analysis in Java. Astrophys. Source Code Libr. Rec. ascl1206.013.
